# Measures to strengthen international biosafety and biosecurity practices

**DOI:** 10.1371/journal.pbio.3003691

**Published:** 2026-03-18

**Authors:** Etienne Decroly, Patrick Berche, Ali Asy, Maggie L. Bartlett, Åsa Szekely Björndal, Christian Brechot, Patrick G. Casey, Patrick Couvreur, Maria J. Espona, Claudia Filippone, Diah Iskandriati, Wilmot James, Rafael Elias Marques, Zabta Khan Shinwari, Bernadett Pályi, Dana Perkins, Kathrin Summermatter, Olivier Vandenberg, Kurt Zatloukal, Jonathan Ewbank

**Affiliations:** 1 Architecture et Fonction des Macromolécules Biologiques (AFMB), Aix-Marseille Université, CNRS, Marseille, France; 2 Department of Microbiology, Université Paris Cité, Paris, France; 3 Animal Health Research Institute, Agriculture Research Center, Giza, Egypt; 4 Johns Hopkins Bloomberg School of Public Health, Johns Hopkins University, Baltimore, Maryland, United States of America; 5 Department of Microbiology, Public Health Agency of Sweden, Stockholm, Sweden; 6 Global Virus Network, University of South Florida, Tampa, Florida, United States of America; 7 Office of the Vice President for Research and Innovation, University College Cork, Cork, Ireland; 8 Institut Galien Paris-Saclay, Université Paris-Saclay, Orsay, France; 9 ArgIQ, Argentina Information Quality, Buenos Aires, Argentina; 10 European Research Infrastructure on Highly Pathogenic Agents, ERINHA AISBL, Brussels, Belgium; 11 Primate Research Center, IPB University, Bogor, Indonesia; 12 Pandemic Center, School of Public Health, Brown University, Providence, Rhode Island, United States of America; 13 Brazilian Biosciences National Laboratory - LNBio, Brazilian Center for Research in Energy and Materials (CNPEM), Campinas, São Paulo, Brazil; 14 Faculty of Biological Sciences, Federal Urdu University of Arts, Sciences and Technology, Karachi, Pakistan; 15 National Biosafety Laboratory, National Center for Public Health and Pharmacy, Budapest, Hungary; 16 Independent Biosecurity Consultant, Washington, DC, United States of America; 17 Institute for Infectious Diseases, University of Bern, Bern, Switzerland; 18 Center for Environmental and Occupational Health, School of Public Health, Université Libre de Bruxelles, Brussels, Belgium; 19 Diagnostic and Research Institute of Pathology, Diagnostic & Research Center of Molecular Biomedicine, Medical University of Graz, Graz, Austria; 20 Institut National de la Santé et de la Recherche Médicale, Paris, France

## Abstract

What concrete steps can we take to reinforce biorisk management immediately? In this Perspective, an international group of experts advocate for robust gatekeeping of funding and publication using a new formal reporting standard for pathogen research.

## Teaser

No matter where they are performed, studies with infectious agents need ironclad biorisk management. This can be promoted through robust gatekeeping of funding and publication, using a new formal reporting standard for pathogen research.

Recent disease outbreaks and epidemics in different parts of the world have provided a stark reminder of the grave threat that microbes represent to public health. There is an absolute necessity for research on so-called ‘pandemic-prone pathogens’ (PPPs), to develop vital medical countermeasures. Studies with these dangerous pathogens must, however, be conducted responsibly and according to strict principles of safety and security.

Determining the appropriate biorisk management strategy requires an evaluation of risk to researchers, the local and general population, and the environment. Thus, when conducting any assessment of risk, one needs to ask how likely an accidental spread of a pathogen from a laboratory is, and what the consequences are likely to be. Over the years, various research-related accidents have been reported [1]. Fortunately, most laboratory-acquired infections are mild because at-risk researchers receive targeted prophylaxis (e.g., through vaccination); however, risks can be higher when dealing with novel pathogens. In one of the most consequential events, in 1967, animal handlers were infected with the then-unknown Marburg virus (*Orthomarburgvirus marburgense*; ICTV19870485; MSL40) following contact with infected monkeys from Uganda [2], leading to 31 infections and 7 deaths. The transmission risk is significantly higher for respiratory viruses, such as Coronaviruses. SARS-CoV (*Betacoronavirus pandemicum*; ICTV20040588; MSL40) has escaped several times from high-containment (biosafety level (BSL)-3) laboratories [3] and once from a maximum-containment (BSL-4) laboratory due to poor oversight, seeding local clusters of infection.

Although biosafety and biosecurity measures have significantly improved over the years, incidents and accidents still happen. They are vanishingly rare in well-managed BSL-4 facilities, but with the increasing number of BSL-3 and BSL-4 laboratories worldwide, risks persist. Furthermore, new genome modification techniques, in combination with advanced in silico methods [4], facilitate the construction of chimeric viruses [5–7], while the de novo synthesis of infectious clones has allowed, for example, the rescue of 1918 H1N1 influenza virus (*Alphainfluenzavirus influenzae*; ICTV19710226; MSL40) [8]. Moreover, the culture of viruses in human cells, organoids, organs, or humanized animal models can introduce the additional risk of selecting viruses with novel tropisms that have enhanced capacity for human infection [9,10].

The legitimacy of such research depends on a framework of transparent governance and proactive communication. The public and policymakers need to be informed about why PPP research is necessary and how risks are mitigated. Because the consequences of a laboratory accident, or of intentional misuse of pathogens, are inherently international, decisions to proceed with high-risk experiments should be taken with a global perspective. Beyond the technical issues, there must be a commitment to ethical oversight that includes diverse perspectives [11]. Two further elements are essential. First, there must be a rigorous assessment of the balance between risks and benefits, even though these are often difficult to anticipate [12]. Such an evaluation is particularly important when dealing with so-called ‘gain-of-function’ research, and as new technologies emerge that facilitate the modification or synthesis of pathogen genomes. Second, for certain categories of experiment known to be particularly hazardous, even in the absence of international regulation, there should be globally agreed biorisk management norms.

Two international standards for biorisk management have been developed (Box 1), but relatively few institutions have implemented either. In the absence of formal common norms, there continue to be vigorous debates about risk assessment and mitigation [13], including in the press. Yet, even without international legally binding norms, best practices can already be promoted by making funding for research conditional on predefined biorisk management mechanisms, and by journals only publishing articles if the work has been conducted responsibly, in conformity with the relevant regulatory framework(s). The recent formulation of the MIHCLE reporting standard [14] (Box 1) provides a semantic framework that can be applied to both. The standard will be particularly useful when applied in conjunction with the new EVORA ontology (Box 1) for describing pathogens. The International Bio Funders Compact (Box 1), which focuses on dual-use research of concern, is also an encouraging advance.

**Box 1. Some examples of biorisk management and reporting initiatives.**

**ISO standards**

Seven years ago, the International Organization for Standardization (ISO) established the ISO 35001:2019 standard on Biorisk management for laboratories and related organizations. It provides, however, little guidance on the quantification of risk or the effectiveness of different risk mitigation measures. It was complemented in 2024 by the publication of ISO/TS 5441:2024, Competence requirements for biorisk management advisors, which covers the knowledge, skills, and experience “needed for an advisor to identify, assess, control, and monitor the risks associated with biological materials”. Collectively, they provide a thorough framework for biorisk management. Substantial long-term investment in human and material resources will be essential if they are to be translated into good practice.

**MIHCLE reporting standard**

The Minimum Information about a High Containment Laboratory Experiment (MIHCLE) reporting standard has been developed for work conducted in BSL-3 and BSL-4 facilities [14]. With 10 sections covering, for example, the type of work performed, and the biosafety and biosecurity practices implemented, it aims to provide a comprehensive, structured description of pathogen research in a manner that increases transparency and supports informed oversight.

**EVORA ontology**

The European Viral Outbreak Response Alliance (EVORA) ontology provides a structured and harmonized vocabulary for describing pathogens and the research that is done with them. It can be used at all stages of the research lifecycle, from project conception onwards. Inherently machine-readable, it represents an inter-operable metadata framework that is fully compatible with the MIHCLE reporting standard.

**International Bio Funders Compact**

The International Bio Funders Compact “incorporates biosecurity into the bioscience research funding process to safeguard life science research from accidental or intentional misuse”. It complements initiatives from individual funding bodies, such as the Coalition for Epidemic Preparedness Innovation’s recently published biosecurity policy.

**WHO actions**

The World Health Organization (WHO) has worked relentlessly for decades to strengthen biorisk management globally, including through the recent adoption of WHA77.7 Agenda item 14.1, Strengthening laboratory biological risk management. It has published a series of reference texts, such as the “Laboratory biosafety manual, 4th edition”, its accompanying practical implementation guide, “Laboratory biosecurity guidance”, and the Global guidance framework for the responsible use of the life sciences: mitigating biorisks and governing dual-use research.

**INTERCEPTOR project**

The INTERCEPTOR project brings together dozens of high and maximum-containment laboratories from around the world. As part of its overall goal to harmonize and strengthen biorisk management, it has developed a dedicated ISO 35001:2019 training program to support laboratories in implementing or improving compliance with the international standard.

Even before journals require submissions to be accompanied by a MIHCLE report, funders could already make committing to the use of the standard a prerequisite of awarding grants for such research. They might even consider requiring applicants to use the standard when submitting their proposals as this could help make oversight more uniform. Journals should also take a more proactive approach to monitoring the conditions under which research with high-consequence pathogens is conducted—regardless of the rules and regulations that might exist in a particular jurisdiction—and reject articles that fail to take a precautionary biorisk management approach. This equally applies to work with emerging pathogens, for which understanding of virulence and pathogenesis may be incomplete, or where no medical countermeasures exist [9, 10].

Given the real risks, we advocate for the harmonization of international biosafety and biosecurity regulations and for improved procedures for risk assessment of pathogen manipulation. Multilateral initiatives such as the International Experts Group of Biosafety and Biosecurity Regulators, and the World Health Organization (WHO) actions aimed at strengthening biorisk management globally (Box 1), must be supported. This is particularly pressing in the context of the loss of US government funding for the WHO, which will have a serious impact on the capacity of low- and middle-income countries to implement effective policies for laboratory biosafety and biosecurity management. It will be important to institute a secure platform for sharing information about accidents, incidents, and near-misses. Steps are being taken towards this within the INTERCEPTOR project (Box 1).

Additionally, we urge researchers and journals to adopt the MIHCLE reporting standard. Journals and their reviewers share an ethical responsibility to ensure that published studies adhere to accepted norms in order to dissuade researchers from working under sub-standard conditions. It will also be crucial to promote education and training in biorisk assessment and management, align biosafety and biosecurity regulations worldwide, and to implement a new type of ‘biological black box’. This would be an integrated system for laboratories researching high-consequence pathogens, serving as a secure data repository to record laboratories’ biorisk assessment processes, as well as for pathogen-related information such as the pathogen sequence and sequencing data from samples of the work environment, to ensure full accountability should accidents occur.

Numerous pathogens are potentially capable of causing epidemics that could spread beyond borders. Be it publicly or privately funded research, we need to move away from accepting risky work with infectious agents just because it followed national guidelines, especially when the guidelines are demonstrably weaker than international norms. We call on regulators, funders, researchers, reviewers, and journals to play their part in promoting the highest possible standards in biorisk management, regardless of where studies are conducted, and so ensure that research vital to public health continues in an accountable, safe, secure, and responsible manner.

## Abbreviations

BSL: biosafety level
EVORA: European Viral Outbreak Response Alliance
ISO: International Organization for Standardization
MIHCLE: Minimum Information about a High Containment Laboratory Experiment
PPPs: pandemic-prone pathogens
WHO: World Health Organization.
